# Identification of microRNAs regulating *Escherichia coli* F18 infection in Meishan weaned piglets

**DOI:** 10.1186/s13062-016-0160-3

**Published:** 2016-11-03

**Authors:** Zhengchang Wu, Weiyun Qin, Seng Wu, Guoqiang Zhu, Wenbin Bao, Shenglong Wu

**Affiliations:** 1 Key Laboratory for Animal Genetics, Breeding, Reproduction and Molecular Design of Jiangsu Province, College of Animal Science and Technology, Yangzhou University, Yangzhou, 225009 People’s Republic of China; 2 College of Veterinary Medicine, Yangzhou University, Yangzhou, Jiangsu People’s Republic of China

**Keywords:** Pigs, microRNA, *E. coli* F18 resistance

## Abstract

**Background:**

*Escherichia coli* F18 is mainly responsible for post-weaning diarrhea (PWD) in piglets. The molecular regulation of *E. coli* F18 resistance in Chinese domestic weaned piglets is still obscure. We used Meishan piglets as model animals to test their susceptibility to *E. coli* F18. Small RNA duodenal libraries were constructed for *E. coli* F18-sensitive and -resistant weaned piglets challenged with *E. coli* F18 and sequenced using Illumina Solexa high-throughput sequencing technology.

**Results:**

Sequencing results showed that 3,475,231 and 37,198,259 clean reads were obtained, with 311 known miRNAs differently expressed in resistant and sensitive groups, respectively. Twenty-four miRNAs, including 15 up-regulated and 9 down-regulated, demonstrated more than a 2-fold differential expression between the F18-resistant and -sensitive piglets. Stem-loop RT-qPCR showed that miR-136, miR-196b, miR-499-5p and miR-218-3p significantly expressed in intestinal tissue (*p* < 0.05). KEGG pathway analysis for target genes revealed that differently expressed miRNAs were involved in infectious diseases, signal transduction and immune system pathways. Interestingly, the expression of miR-218-3p in intestinal tissue had a very significant negative correlation with target *DLG5* (*P* < 0.01).

**Conclusions:**

Based on the expression correlation between miRNA and target genes analysis, we speculate that miR-218-3p targeting to *DLG5*, appears to be very promising candidate for miRNAs involved in response to *E. coli* F18 infection. The present study provides improved database information on pig miRNAs, better understanding of the genetic basis of *E. coli* F18 resistance in local Chinese pig breeds and lays a new foundation for identifying novel markers of *E. coli* F18 resistance.

**Reviewers:**

This article was reviewed by Neil R Smalheiser and Weixiong Zhang.

**Electronic supplementary material:**

The online version of this article (doi:10.1186/s13062-016-0160-3) contains supplementary material, which is available to authorized users.

## Background

MicroRNAs (miRNAs) are approximately 22 nucleotides long, highly conserved non-coding RNAs (ncRNAs) encoded in the genome of all eukaryotes. These miRNA molecules have resulted from translational repression or deadenylation of target mRNA by binding complementary target sites in the 3′untranslated region (3′UTR) of mRNA [1]. It was noticed early that the 5′ end of miRNAs is important for the binding of target mRNAs, in particular nucleotides 2–8, which is sometimes referred to as the ‘seed’ [2]. In animals, a few miRNAs can regulate gene expression at the post-transcriptional level by encoding target protein mRNAs involved in cellular growth, differentiation, proliferation, apoptosis and immune response [3, 4]. However, unknown functional roles of miRNAs still remain to be elucidated.

To date, the discovery and functional verification of a majority of miRNAs have been implemented in human and model animals. Compared to the human miRNAs, the porcine miRNA studies have seriously fallen behind. The number of annotated miRNAs in domestic pig in the newest miRBase 20.0 (http://www.mirbase.org/) is still much smaller than those in other organisms, featuring 1872 precursor and 2578 mature miRNAs for human, with only 280 precursor and 326 mature for pig by 2015. Initially, porcine miRNAs were discovered by the means of a homology search. In recent years, with the emergence of Solexa and 454 high-throughput sequencing technologies, these make it possible for obtaining new porcine miRNAs by direct sequencing. Many studies focused on the role of porcine miRNAs in the viral diseases, such as porcine reproductive and respiratory syndrome (PRRS) and H1N1 influenza in pigs [5–7], especially played a role in regulating the immune responses [8–10]. Importantly, the ability of miRNAs to regulate gene expression and their stability make them useful tools to guide breeding programs for porcine disease resistance in the livestock field.

Enterotoxigenic *Escherichia coli* (ETEC) is an important pathogenic bacteria causing severe diarrhea in humans and pigs. Previous studies have confirmed that the *E. coli* F18 strain is the main pathogen responsible for porcine post-weaning diarrhea (PWD) [11]. Via its fimbriae, ETEC F18 pathogen adheres to the surface of epithelial cells of the small intestines of piglets and binds to specific receptors in the brush border membrane host intestinal mucosa, leading to colonization, replication and production of enterotoxin and lipopolysaccharide (LPS) [12]. Therefore, resistance to *E. coli* F18 depends on expression of receptors on alvine epithelial cells and individual immunity. Previous studies demonstrated that a G to A mutation at locus M307 of alpha (1,2)fucosyltransferase gene (*FUT1*) could control the expression of the *E. coli* F18 receptor, which has been proposed to be a candidate gene for the selective breeding of *E. coli* F18 adhesion-resistant pigs [13, 14]. However, the *FUT1* M307 locus displays a polymorphism only in foreign pig breeds, such as Duroc, Pietrain, Yorkshire and in hybrid lines bred with foreign lineages, such as the Sutai pig. Chinese domestic pig breeds, however, except the Lingao breed that carries an AG genotype, do not have the AA genotype or even AG genotype [15–17]. Therefore, the *FUT1* M307 marker is not suitable for Chinese domestic breeds, suggesting that Chinese and foreign pig breeds have different molecular mechanisms and physiological functions related to the formation and structure of receptor molecules or innate and adaptive immunity against *E. coli* F18 infection, which makes breeding of resistant Chinese domestic breeds difficult. Therefore, it is necessary to seek effective molecular markers for *E. coli* F18 resistance in Chinese domestic pig breeds.

However, it is difficult to analyze the molecular mechanism of *E. coli* F18 resistance using high-throughput sequencing technology in Chinese domestic pig breeds because of the lack of extreme phenotype individuals for *E. coli* F18 infection. In the present study, Meishan piglets were used as model animals to test their susceptibility to *E. coli* F18 by challenging with F18 strains. After a series of experiments, such as *E. coli* F18 bacteria detection, bacteria counting and adhesion test of the pathogens to the epithelial cells of small intestine in vitro, we strictly identified *E. coli* F18-resistant and -susceptible complete sib-pair individuals [18]. Solexa high-throughput sequencing technology is a convincing strategy for identifying miRNAs. To assess the effects and mechanism of differentially expressed miRNAs for the resistance to *E. coli* F18, we compared the duodenal miRNA transcriptomes of resistant and sensitive piglets to *E. coli* F18 using Solexa high-throughput sequencing technologies. Thus, our study will provide a thorough investigation of the miRNAome in porcine duodenum to facilitate a better understanding of the resistance mechanism to *E. coli* F18 in Chinese domestic weaned piglets.

## Results

### Overview of sequencing data

**Fig. 1:**
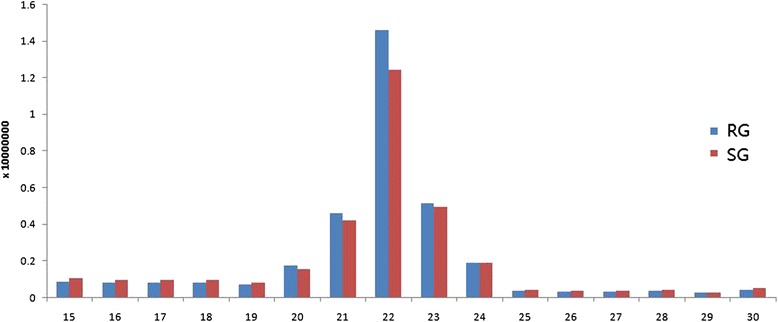
Length distribution for total sRNA reads from the two libraries (RG and SG). RG represents *E. coli* F18-resistant individuals. SG represents *E. coli* F18-sensitive individuals

**Fig. 2:**
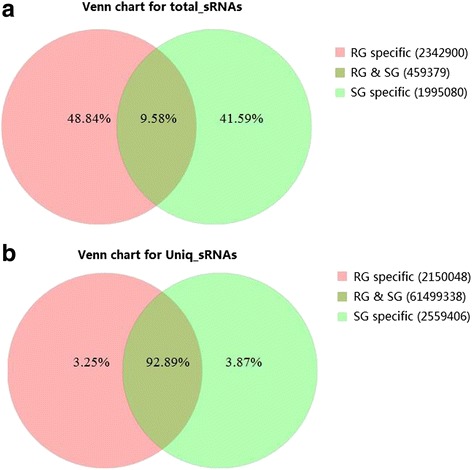
Comparisons of total sRNA reads (**a**) and unique sRNA reads (**b**) from the two libraries. The overlapping sector shows common sequences; the other sectors show the respective specific sequences

To identify miRNAs in *ETEC* F18 infection in Meishan piglets, two small RNA libraries from F18-resistant groups (RG, *n* = 3) and -sensitive groups (SG, *n* = 3) were constructed, respectively, and sequenced using the Illumina-HiSeq 2000 sequencing platform. As a result, a total of 72,505,226 and 62,837,064 raw reads were identified in RG and SG groups, respectively. After removing the low quality reads, ultimately a total of 43,475,231 and 37,198,259 clean reads ranging in size from 15 nt to 30 nt were retrieved from the RG and SG libraries, respectively. The size distribution of the clean reads is shown in Fig. 1. Interestingly, the size distribution of the miRNAs was similar between the small RNA libraries of the *ETEC* RG and SG piglets. The number of 21–23 nt sequences (63.58 %) was significantly greater than that of shorter or longer sequences. Almost half of the sequences in the RG and SG libraries were 22 nt, which is consistent with the known specificity of Dicer processing and the features of mature miRNAs. Comparison of the total sRNA reads and unique sRNA reads indicated that a large percentage of the unique sRNA reads were common to both libraries, whereas the library-specific reads/sequences accounted for only 3.25 to 3.87 % of the unique sRNA reads. In contrast, only 9.58 % of the total sRNA common sequences were common to both libraries, and most of the total sRNA reads were library-specific (Fig. 2).

**Fig. 3:**
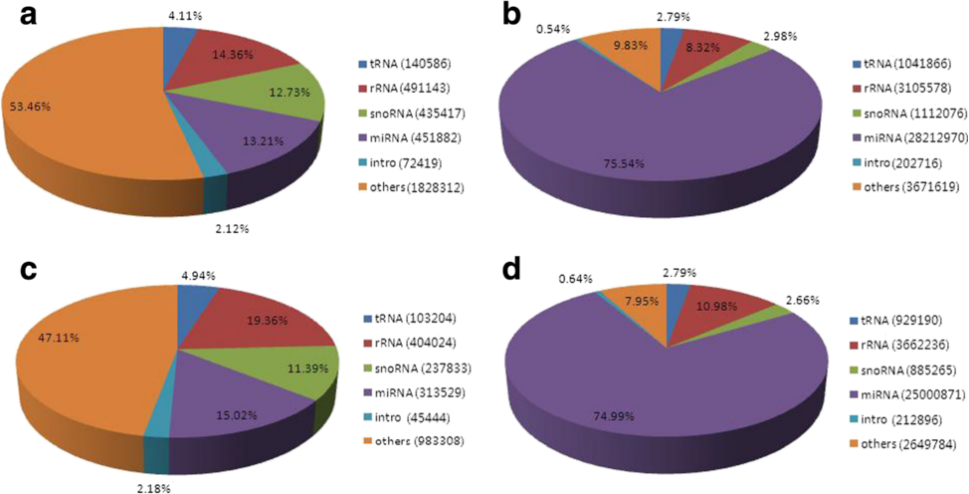
Composition of small RNA classes of Illumina-HiSeq sequencing. **a** Total number of unique sequences in the F18-resistant (RG) library. **b** Total number of reads in the F18-resistant (RG) library. **c** Total number of unique sequences in the F18-sensitive (SG) library. **d** Total number of reads in the F18-sensitive (SG) library

For assessing the efficiency of Illumina-HiSeq sequencing and the quality of sequence itself, all of the clean reads were annotated and classified by aligning against the Rfam10.1 database, GenBank and the miRBase20.0 database. In the present study, all of the clean reads were divided into the following categories: tRNA, rRNA, snoRNA, miRNA, intro and others. As shown in Fig. 3, conserved miRNAs accounted for 75.54 and 74.99 % of the total clean reads in the RG and SG small RNA libraries, respectively. Additionally, conserved miRNAs accounted for 13.21 and 15.02 % of the unique reads in the RG and SG small RNA libraries, respectively. The majority of total reads were classified as miRNA, suggesting that the sequencing of the present study was successful.

### Differential miRNAs between *E. coli* F18-sensitive and -resistant Meishan piglets

**Fig. 4:**
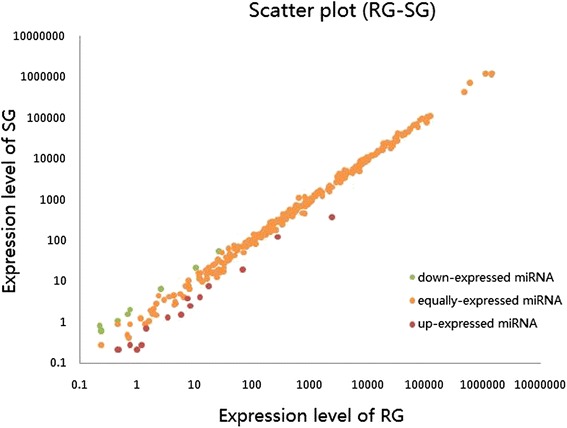
Differential expression of conversed miRNAs between RG and SG library. Each point in the figure represents a miRNA. Red points represent miRNAs with fold-change > 2, orange points represent miRNAs with fold-change > 1/2 and ≤ 2, and green points represent miRNAs with fold-change ≤ 1/2

After successive filtering of these data sets, we observed a total of 311 known miRNAs (Additional file 1) in both libraries (RG and SG). Among three hundred and eleven miRNAs, 15 up-regulated and 9 down-regulated (Fig. 4; Additional file 2), were found to have more than a 2-fold differential expression between the RG and SG piglets. In addition, 681 potential novel miRNA candidates were obtained from RG and SG libraries. These pre-miRNAs possessed a typical stem-loop structure and free energy ranging from –64.8 Kcal/mol to –18.2 Kcal/mol (Additional file 3). The folding structures of partial miRNA precursors (free energy > 50.0 Kcal/mol) are shown in Additional file 4.

### Validation of miRNA expression with stem-loop qRT-PCR

**Fig. 5:**
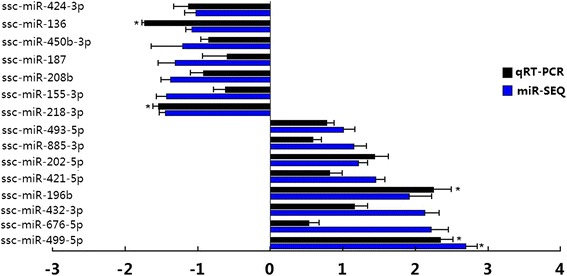
RT-PCR validation of miRNAs identified in Meishan piglets using miRNA sequencing technology. The abscissa shows the value of log_2_ (fold change). Fold change means *E. coli* F18-resistant group/*E. coli* F18-sensitive group. *indicates the significant (*P* < 0.05) difference in expression level between F18-sensitive and F18-resistant piglets by GLM of the SPSS 18.0 software

To validate the reliability of the sequencing data, we conducted RT-qPCR to compare the expression levels of the differentially expressed miRNAs. The expression levels of 15 selected known miRNAs were verified in the duodenum of F18-resistant and sensitive piglets using RT-qPCR. The relative expression levels of most of the 15 selected miRNAs were consistent with the Illumina sequencing results (Fig. 5, Table 1). Interestingly, RT-qPCR showed that ssc-miR-136 and ssc-miR-218-3p were significantly up-regulated in F18-sensitive piglets (*P* < 0.05), and ssc-miR-196b and ssc-miR-499-5p were significantly up-regulated in F18-resistant piglets (*P* < 0.05).

**Table 1:** Validation of the miR-SEQ expression profiles of selected miRNAs by qRT-PCR

miR-name	Accession No.	*E. coli* F18-resistant group	*E. coli* F18-sensitive group	*P*-value
ssc-miR-499-5p	MIMAT0013877	2.370 ± 1.109	0.467 ± 0.055	0.041*
ssc-miR-676-5p	MIMAT0017382	1.890 ± 1.181	0.650 ± 0.294	0.163
ssc-miR-432-3p	MIMAT0017384	1.830 ± 1.344	0.821 ± 0.563	0.215
ssc-miR-196b	MIMAT0013923	2.275 ± 0.991	0.478 ± 0.079	0.036*
ssc-miR-421-5p	MIMAT0017970	0.593 ± 0.183	0.378 ± 0.011	0.127
ssc-miR-202-5p	MIMAT0013948	1.878 ± 1.322	0.695 ± 0.332	0.133
ssc-miR-885-3p	MIMAT0013903	1.284 ± 0.492	0.859 ± 0.269	0.180
ssc-miR-493-5p	MIMAT0025377	1.397 ± 0.680	0.814 ± 0.154	0.146
ssc-miR-218-3p	MIMAT0017969	0.649 ± 0.443	1.901 ± 0.553	0.012*
ssc-miR-155-3p	MIMAT0017953	0.817 ± 0.442	1.659 ± 0.823	0.280
ssc-miR-208b	MIMAT0013912	0.808 ± 0.408	1.538 ± 0.727	0.130
ssc-miR-187	MIMAT0020587	0.815 ± 0.508	1.404 ± 0.044	0.231
ssc-miR-450b-3p	MIMAT0017380	0.427 ± 0.127	0.559 ± 0.112	0.171
ssc-miR-136	MIMAT0002158	0.582 ± 0.224	1.939 ± 0.850	0.045*
ssc-miR-424-3p	MIMAT0013921	0.742 ± 0.234	1.625 ± 0.926	0.215

*p < 0.05

### miRNA target gene prediction, GO enrichment and KEGG pathway analysis

To better understand the biological function of the 15 up-regulated and 9 down-regulated miRNAs (fold-change > 2) in the F18-resistant piglets compared with the F18-sensitive piglets, their target genes were predicted using miRanda database (http://www.microrna.org/microrna/home.do). A total of 87,715 target sites in 12,024 target genes were predicted for 24 differential miRNAs (Additional file 5). GO enrichment analysis was used on the target gene candidates of differentially expressed miRNAs. As shown in Additional file 6, GO enrichment analysis of differentially expressed miRNAs from cellular components showed that 10,933 genes were termed as cellular component ontology with a *P* < 0.01. Analysis of biological processes showed 7,367 genes were involved in anatomical structure development, signal transduction, cell adhesion and cytoskeleton organization (*P* < 0.01). Analysis of the molecular function category showed 5,493 genes were related to cytoskeletal protein binding, ion binding and transcription factor binding (*P* < 0.01).

**Fig. 6:**
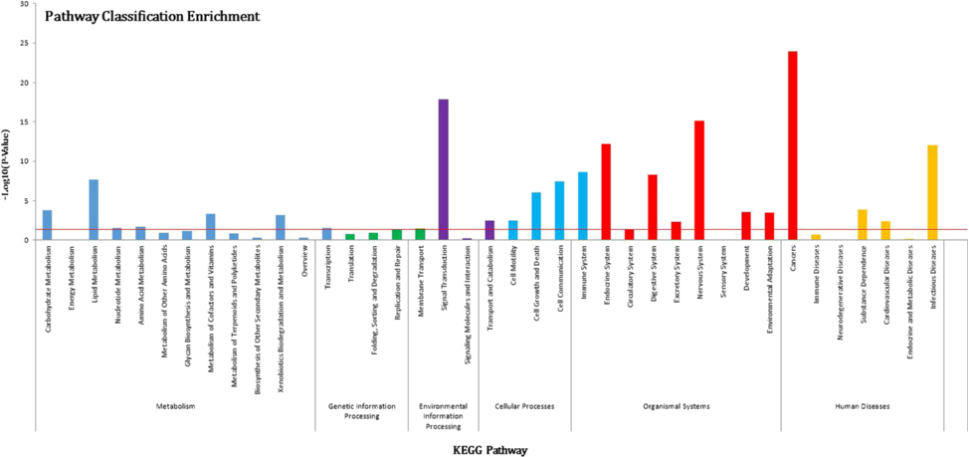
KEGG pathway classification annotated by DAVID for target genes of differentially expressed miRNAs. The figure shows partial KEGG enrichment for the predicted potential target mRNAs in metabolism, genetic information processing, environmental information processing, cellular processes, organismal systems and human diseases

**Fig. 7:**
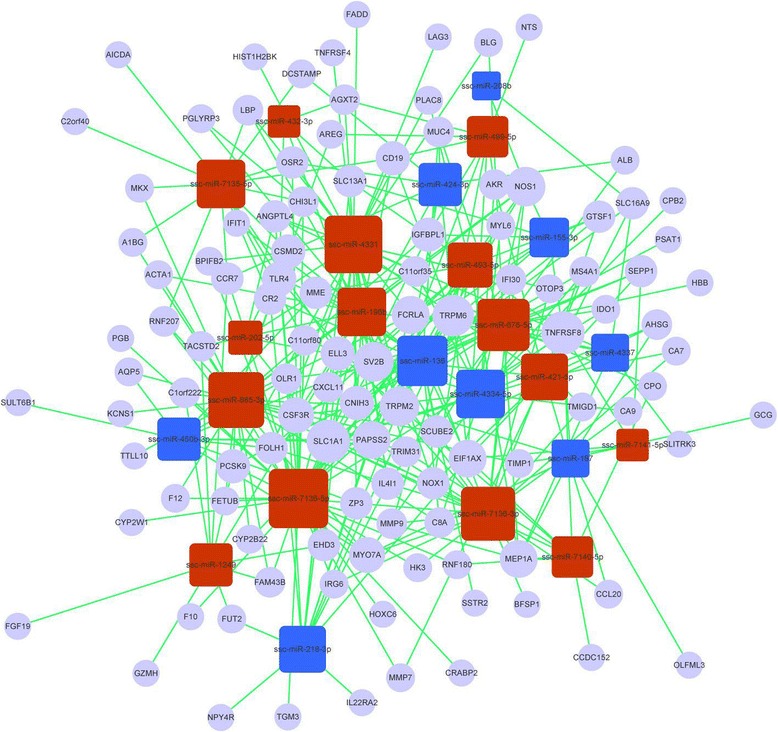
Network analysis of differential expressed miRNAs interacting with potential target genes related to *E. coli* F18 infection. Blue box represents down-regulated miRNAs in the *E. coli* F18-resistant group compared with the *E. coli* F18-sensitive group. Red box represents up-regulated miRNAs in the *E. coli* F18-resistant group compared with the *E. coli* F18-sensitive group

The predicted target genes of 24 differential miRNAs were further classified to identify pathways according to KEGG functional annotations (Fig. 6). KEGG pathway analysis for the target genes revealed that differentially expressed miRNAs were mainly involved in infectious diseases, signal transduction and immune system pathways (Table 2). Based on previous DGEs and functional enrichment, we further screened out important target genes related to *E. coli* F18 infection by Venny software (http://bioinfogp.cnb.csic.es/tools/venny/) and analyzed the regulatory network between important target genes and differential miRNAs (Fig. 7). Based on our results, we speculate that the α-(1, 2) fucosyltransferase 2 gene (*FUT2*) and Discs, large homolog 5 (*DLG5*) genes were the targets of down-regulated ssc-miR-218-3p, the *MUC4* gene was the target of down-regulated ssc-miR-136, *MyD88* was the target of up-regulated ssc-miR-499-5p, *LBP* and Toll-like receptor (*TLR4*) genes were the target of up-regulated ssc-miR-196b.

**Table 2:** Partial pathway annotation

Pathway	Target genes	*p*-Value	-Log10(*P*-Value)
Infectious diseases	1078 (11.67 %)	8.73E-13	12.05922779
Signal transduction	1063 (11.51 %)	1.30E-18	17.88541603
Cancers	795 (8.61 %)	1.26E-24	23.90091113
Immune system	629 (6.81 %)	2.29E-09	8.640783591
Endocrine system	532 (5.76 %)	6.76E-13	12.16987802
Nervous system	346 (3.75 %)	6.95E-16	15.15809019
Transport and catabolism	260 (2.82 %)	3.73E-03	2.42850837
Carbohydrate metabolism	252 (2.73 %)	1.55E-04	3.80968035

### Validation of important target genes expression with qRT-PCR

**Fig. 8:**
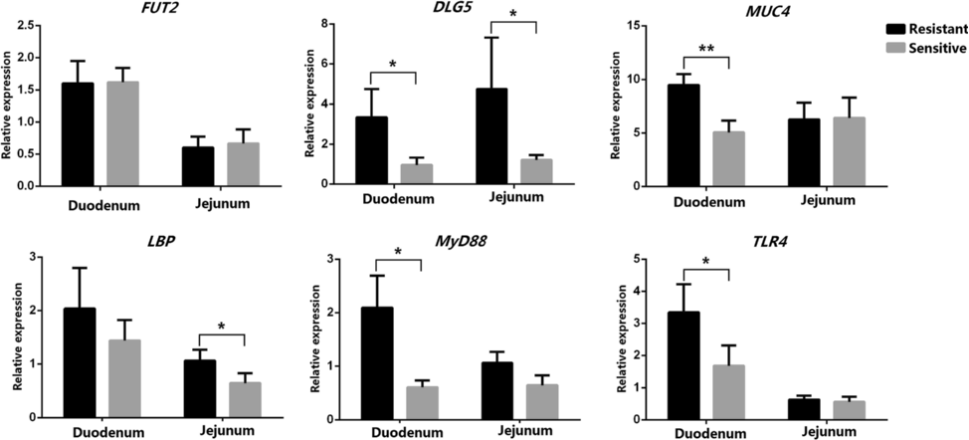
Expression of several potential target mRNAs in intestinal tissues of *E. coli* F18-resistant and -susceptible piglets. *means the difference was significant in the test level of *P* < 0.05 and **means extremely significant in 0.01 level

Expression levels of several target genes of interest were compared in duodenal and jejunal tissues of *E. coli* F18-sensitive and resistant piglets. Detailed analysis of the results is shown in Fig. 8. The expression level of *DLG5* in duodenal and jejunal tissues of the resistant group was significantly higher than that in the sensitive group (*P* < 0.05), and the expression level of the *MUC4* gene was significantly higher in duodenal tissues of resistant pigs (*P* < 0.01). *LBP*, *MyD88* and *TLR4* also showed significantly higher expression in the resistant group (*P* < 0.05).

### Comparison of the expression levels of target genes in duodenal and jejunal tissues

**Fig. 9:**
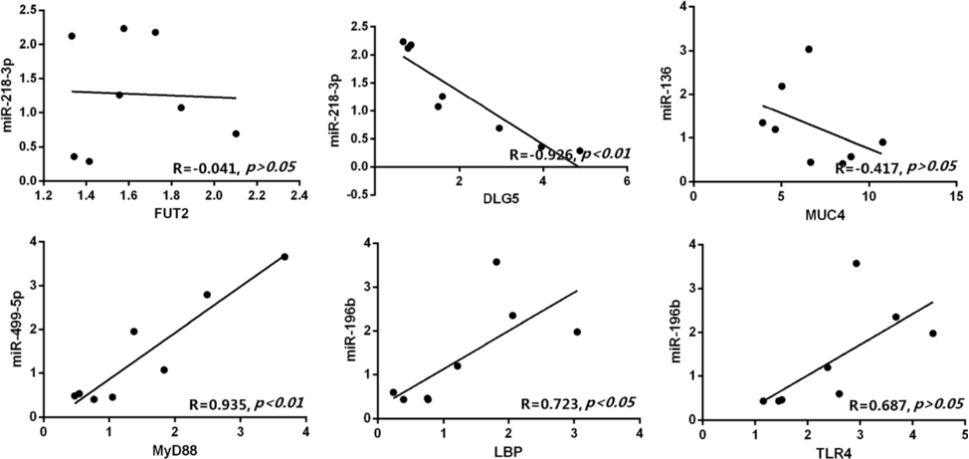
Correlation analysis of gene expression of miR-218-3p with six potential target mRNAs in intestinal tissue. *n* = 8, R_0.05_ = 0.707, R_0.01_ = 0.834. If R-value is higher than R_0.05_ or R_0.01_, the correlation coefficient was significant or extremely significant

In this study, we analysed the correlation between expression of miR-218-3p and six target genes (Fig. 9). The expression level of miR-218-3p in intestinal tissue had a very significant negative correlation with *DLG5* (*P* < 0.01), but there were no significant correlations with *FUT2*, *MUC4* and *TLR4* expression. However, the expression level of the miR-218-3p had significant positive correlations with *LBP* and *MyD88* expression (*P* < 0.05). These results suggested that *DLG5* was probably an important target gene of miR-218-3p.

## Discussion

Piglets are most susceptible to diarrhea disease caused by *E. coli* F18 infection at weaning time. As previously mentioned, foreign breeders have demonstrated that the *FUT1* M307 locus is a genetic marker for breeding resistance to *E. coli* F18 in large white pigs. However, there are some differences in the genetic basis of resistance to *E. coli* F18 infection between Chinese and foreign breeds [15–17]. We previously established the Sutai pig (a new hybrid between the Duroc and Meishan breeds) population that are resistant and sensitive to *E. coli* F18 through selection and assortative mating of AG-type (*FUT1*) Sutai pigs [15]. On this basis, we further analyzed differential gene expression patterns, important pathways and miRNAs between Sutai pig resistant and sensitive to *E. coli* F18 by microarray and Solexa sequencing technology (data available at Gene Expression Omnibus, Accession number: GSE26854, GSE32527). Nevertheless, the Sutai pig with foreign Duroc DNA cannot completely reveal the regulatory mechanism of *E. coli* F18 resistance in Chinese domestic pig breeds. Taihu pig is one breed with the highest reproductive traits in Chinese and foreign pigs around the world. Thereinto, Meishan is the most prominent representative population of Taihu pigs and has the advantage of higher litter size, more delicious meat, etc. Furthermore, compared to commercial western pig breeds, the Chinese Meishan pigs exhibit not only the higher litter size but also an increased physiological maturity which is correlated with the piglet survival rate before and at birth. For Meishan pigs, no suitable genetic markers, including the *FUT1* gene, could be applied to screen F18-resistance and -sensitive individuals, so the current study attempted to use piglets to conduct the bacterial infection experiment. Through summarizing and studying previous experiences, this study seriously considered the following points. Firstly, we detected rotavirus and *E. coli* (F18, K88) in piglet feces before the challenge experiment, excluding experimental piglets that carried the rotavirus and/or *E. coli*. Secondly, this study specially prepared piglet feed without antibiotics and probiotics, avoiding an adverse impact on the experiment. Thirdly, the challenge experiment was verified and validated by *E. coli* F18 bacteria detection, bacteria counting and adhesion of small intestinal epithelial cells. Similar to our previous study above, we improved the effectiveness and feasibility of our piglet diarrhea model using an artificial challenge experiment.

In recent years, high-throughput sequencing has become a powerful strategy for identifying novel miRNAs and studying the expression profiles of miRNA in different samples. Unlike microarray technology, Solexa high-throughput sequencing sheds light on functionally novel miRNAs [19, 20]. In this study, we obtained *E. coli* F18-resistance and -sensitive Meishan weaned piglets, and discovered 311 known miRNAs and 681 novel miRNAs in the duodenum by Illumina Solexa technology. The intestinal tract is the first line of defense against infection and the main place where pathogenic microorganisms colonize and replicate. A healthy intestinal microbiota, a microbial community consisting of eukaryotes, viruses and bacteria, is essential for the health of the host and provides protection against enteric infection [21]. According to reports, miRNAs control intestinal cell differentiation, physiology, barrier function and cellular apoptosis [22, 23]. Furthermore, miRNAs are involved in the pathogenesis of intestinal cancer, inflammatory bowel disease, irritable bowel syndrome and cystic fibrosis, among others [24–28]. At present, there are relatively few reports of piglet intestinal miRNA sequencing. Sharbati et al. (2010) conducted miRNA cDNA library sequencing on six different sections (duodenum, anterior segment of jejunum, posterior segment of jejunum, ileum, ascending colon and transverse colon) of 31-day-old healthy piglet (EUROC × Pietrain) intestinal tissues and found that miR-194 and miR-215 were highly expressed in the duodenum and posterior segment of the jejunum and that miR-19b, miR-23a, miR-24 and miR-30b expression was higher in the colon than in other sections of the intestinal tract [29]. Tao et al. (2013) conducted miRNA sequencing on jejunum tissue of newborn litters of crossbred piglets (DYL, originating from mating Duroc boars with Yorkshire-Landrace sows) and found that the expression of miR-215 and miR-146b were significantly different at different days (*P* < 0.05) [30]. Ye et al. (2012) conducted miRNA sequencing on duodenum tissues of 28-day-old Sutai (Duroc × Meishan) resistant and sensitive to *E. coli* F18 piglets and identified 12 candidate miRNA disease markers, especially the expressions of miR-215 and miR-192 were found to be significantly different between *E. coli* F18-sensitive and -resistant groups [31].

In the present study, RT-qPCR showed that the expression of miR-136, miR-218-3p, miR-196b and miR-499-5p were significantly up- or down-regulated between F18-sensitive and -resistant piglets. Previous studies have shown that above miRNAs (miR-136, miR-218-3p, miR-196b and miR-499-5p) indeed play roles in the development and regulation of human disease [32–35]. To gain further insight into the physiological function of the above four miRNAs in the resistance to F18 infection in pig intestinal tract, we predicted and screened out potential target mRNAs, including *FUT2*, *DLG5*, *MUC4*, *TLR4*, *MyD88* and *LBP*. Coddens et al. demonstrated that the minimal binding epitope of the F18 receptor is the blood group H type 1 determinant (Fucalpha2Galbeta3GlcNAc), and *FUT2* catalyzes the formation of blood group H type 1 [36]. *DLG5* belonging to the membrane-associated guanylic acid kinase family, located at the cell junction and plays a critical role in regulating cell growth, maintaining the structural integrity of epithelial cells and signal transmission [37]. The intestinal mucosa integrity of epithelial cells is an important structural foundation of the resistance to *E. coli* F18 infection. Moreover, *MUC4* is a trans-membrane member of the mucin family, which protects and lubricates epithelial surfaces. It has been previously reported that a genetic variation of the *MUC4* gene is associated with susceptibility/resistance to *ETEC* F4 infection [38]. *LBP*, *TLR4* and *MyD88* are key genes involved in in the TLRs pathway. The TLR family recognizes conserved microbial structures, such as bacterial lipopolysaccharide and viral double-stranded RNA, and activates signaling pathways that result in immune responses against microbial infections [39]. The TLRs sense microbial populations in the intestine and initiate proinflammatory signaling pathways against invading microbial pathogens [40]. In our study, the expression level of miR-218-3p in intestinal tissue had a very significant negative correlation with *DLG5* (*P* < 0.01). Therefore, we speculated that miR-218-3p targeting *DLG5* was likely to regulate the formation of the *E. coli* F18 receptor and maintain the intestinal mucosa integrity of epithelial cells. Meanwhile, *MyD88, LBP* and *TLR4* probably play an important role in stimulating immune and antigen presentation in piglet’s response to the infection of *E. coli* F18.

In future research, we will examine the targeted relationship between candidate miRNAs and key targets using a luciferease assay. Furthermore, we will integrate overexpression and RNA interference of our candidate miRNAs in piglet intestinal epithelial cell lines as well as the type V secretion system and receptor binding experiments. In addition, an *E. coli* F18 infection experiment will be established for functional analysis of potential target mRNAs. These studies will further our understanding of the mechanisms of miR-218-3p as well as miRNA-mediated genes in the regulation of Mershan weaning piglets resistance to *E. coli* F18 and lay a solid foundation for the breeding of disease resistance to *E. coli* in Chinese domestic pig breeds.

## Conclusions

In conclusion, we initially identified miR-196b, miR-499-5p and miR-218-3p as candidate miRNAs involved in *E. coli* F18 infection by miRNAs sequencing and qRT-PCR validation. Potential target mRNAs of differently expressed miRNAs were mainly involved in infectious diseases, signal transduction and immune system pathways. Based on the expression correlation between miRNA and potential target mRNAs, we speculate that *DLG5*, potential target gene of miR-218-3p, probably acts as a novel marker of *E. coli* F18 resistance.

## Methods

### Ethics statement

The animal study proposal was approved by the Institutional Animal Care and Use Committee (IACUC) of the Yangzhou University Animal Experiments Ethics Committee with the permit number: SYXK(Su) IACUC 2012-0029. All piglet experimental procedures were performed in accordance with the Regulations for the Administration of Affairs Concerning Experimental Animals approved by the State Council of People’s Republic of China.

### Challenge experiment with *E. coli* F18 strain and sample collection

Meishan weaning piglets were collected from Kunshan Conservation Ltd. (Suzhou City, Jiangsu Province, China). We selected three litters of weaning piglets at 35 days of age, 12 piglets with almost same birth weight and weaning weight per litter. Twelve piglets per litter were randomly divided into two groups: the control group (two piglets) and the experimental group (ten piglets). Each piglet was housed individually in separate pens and fed ad libitum with a commercial-type compound feed for weaned piglets containing 21.7 % crude protein, without antimicrobial additives and organic acids. Beginning at day 3 post-weaning, experimental piglets were challenged with a daily dose of 4.6 × 10^8^ CFU of *E. coli* F18 strain once a day for up to 10 days, or until they showed diarrhea. No additional food was given and we ensured that piglets ate all food before the challenge experiment. Throughout the experiment, fecal shedding of the inoculated bacteria was monitored by daily fecal sampling and feces consistency was scored using the parameters “normal”, “pasty” and “watery”. Only piglets with watery feces were considered as diarrheic. The intestinal tracts of the diarrheic pigs were used to carry out a series of experiments, such as *E. coli* F18 bacteria counting, histopathological detection and adhesion test of the pathogens to the epithelial cells of small intestine in vitro [41]. Nine piglets showing “watery diarrhea” were defined as the “diarrhea group” and eight normal piglets as the “normal group”. After slaughter, we took intestinal tissues (duodenum, jejunum and ileum) to detect bacterial numbers. In all piglets detected by the binding assay, we strictly identified piglets displaying no adherence with F18-expressing fimbriae of the standard *ETEC* strain as *E. coli* F18-resistant individuals (Additional file 7A). In contrast, piglets displaying a large amount of adherence were identified as*E. coli* F18-susceptible individuals (Additional file 7B and C). According to the above method, we selected three resistant and three sensitive piglets to *E. coli* F18 for miRNA sequencing. About 100 mg of duodenal tissue was removed and the scraped epithelium of the duodenum was placed into 1.5 mL nuclease-free Eppendorf tubes, frozen in liquid nitrogen and stored at –80 °C until further use.

### Preparation of small RNA library and Solexa sequencing

Six small RNA libraries were constructed from resistant (R1, R2, R3) and sensitive (S1, S2, S3) piglets to *E. coli* F18. Total RNA was extracted from the duodenum of resistant and sensitive piglets using Trizol reagent (Invitrogen Life Technologies, USA) in accordance with the manufacturer’s protocol. RNA degradation and contamination was monitored on 1 % agarose gels. RNA integrity was assessed using the RNA Nano 6000 Assay Kit of the Bioanalyzer 2100 system (Agilent Technologies, CA, USA). For each group, a total amount of 4 μg total RNA per sample was used as input material for the small RNA sample preparations. Sequencing libraries were generated using NEBNext® Multiplex Small RNA Library Prep Set for Illumina® (NEB, New England Biolab, Ipswich, MA, USA.) following manufacturer’s recommendations. Index codes were added to attribute sequences to each sample. Briefly, NEB 3′ SR Adaptor was directly, and specifically ligated to the 3′ end of the miRNA; after the 3′ ligation reaction, the SR RT primer was hybridized. cDNA constructs were created by RT-PCR based on the small RNAs ligated with 39 and 59 adaptors. The PCR products (155 bp, small RNA + adaptors) were purified with 0.8 % agarose gels and used for sequencing with Solexa sequencing technology (Illumina HiSeq 2000, Shanghai Personal Biotechnology Cp., Ltd. Shanghai, China).

### Sequencing data analysis

Raw data (raw reads) in fastq format were firstly processed through custom perl and python scripts. In this step, the clean data (clean reads) were obtained by removing reads containing ploy-N, with 5′ primer contaminants, without 3′ primers or the insert tag, containing ploy A, T, G or C and low quality reads from raw data. The clean reads were mapped to the *sus scrofa* genome without mismatch to analyze their expression and distribution using Bowtie software [42].

Mapped small RNA tags were used to looking for known miRNA. The sequences were aligned against the known miRNAs precursors and mature miRNAs deposited in the miRBase 20.0 to identify conserved miRNAs. The characteristics of the hairpin structure of the miRNA precursor can be used to predict novel miRNA. Custom scripts were used to obtain the miRNA counts as well as base bias on the first position of identified miRNA with certain length and on each position of all identified miRNA, respectively. To remove tags originating from protein-coding genes, repeat sequences, rRNA, tRNA, snRNA and snoRNA, small RNA tags were mapped to RepeatMasker, Rfam or those types of data from the specified species itself. To identify novel miRNA genes among the unannotated sequences in our libraries, we employed the mireap program (https://sourceforge.net/projects/mireap/), which processes high-throughput sequencing data sets. The RNAfold software in the ViennaRNA Package 2.0 [43] was also used to predict the typical secondary structures of the miRNA precursors.

### Analysis of differentially expressed miRNAs

Expression levels of all miRNAs were quantified by FPKM values using the Cufflink software [44]. Differential expression levels of miRNA between the F18-resistant group (RG) and sensitive group (SG) were assessed by the normalization method: (1) Normalized expression (NE) = Actual miRNA count/Total count of clean reads × 1,000,000; (2) Calculate fold-change from the normalized expression. The differential expression of miRNAs in the two groups were analyzed by DESeq (http://bioconductor.org/packages/release/bioc/html/DESeq.html) and ultimately shown by plotting Log2-ratio figure and scatter plot.

### miRNA validation via stem-loop qPCR

The stem-loop RT-qPCR method was used to validate differentially expressed miRNAs [45]. Total RNA was reverse-transcribed to cDNA in a total volume of 10 μL using Takara PrimeScript RT reagent kit (Takara, Dalian, China). The stem-loop quantitative real-time PCR was performed on an ABI 7500 system (Applied Biosystems, Foster City, CA, USA). Porcine U6 snRNA was used as a housekeeping gene, and all reactions were run in triplicate. The miRNA-specific stem-loop RT primers were designed (Additional file 8) and synthesized with the software primer 5.0.

### Predicted target genes and functional annotation

In the present study, potential target mRNAs of identified known differentially expressed miRNAs (DEMs) between the resistant and sensitive groups were predicted using the online database miRecords [46] (http://mirecords.biolead.org/). To gain further insight into the biological functions of the identified miRNAs, we performed a Gene Ontology (GO) term and KEGG pathway annotation of the predicted miRNA targets using the DAVID gene annotation tool (http://david.abcc.ncifcrf.gov/). We used the KOBAS software [47] to test the statistical enrichment of potential target mRNAs in GO and KEGG pathways. According to previous differential expression genes (DEGs) between Meishan F18-resistant and -sensitive groups by transcriptome sequencing (data are available at NCBI’s SRA, PRJNA271310), we further selected potential target mRNAs related to *E. coli* F18 infection. On this basis, the regulatory network of DEMs and potential target mRNAs was established by Cytoscape software [48].

### qRT-PCR validation of target genes related to *E. coli* F18 infection

Total RNA was extracted using the TRIzol Reagent (Takara Biotech Co., Ltd., Dalian, China), according to the manufacturer’s instructions. The housekeeping genes *TBP1*, *ACTB* and *GAPDH* were used as a reference control to normalize the expression level. All primers of potential target mRNAs and housekeeping genes were shown in Additional file 9. We performed qPCR using an ABI 7500 system (Applied Biosystems, Foster City, CA, USA). Each reaction volume contained 10 μl 2× SYBR Premix Ex *Taq* II (Takara, Dalian, China), 0.4 μl forward primer (10 μM), 0.4 μl reverse primer (10 μM), 0.4 μl 50× ROX Reference Dye II (Takara, Dalian, China), and 2 μl cDNA, and ddH_2_O to 20 μl.

### Statistical analysis

The comparative CT method (2^-∆∆CT^ method) [49] was used to analyze the relative expression level of differential expression miRNAs and potential target mRNAs. The general linear model (GLM) was carried out to determine the significance of differences in mRNA relative expression between the resistant and sensitive groups. Correlation analysis was performed pairwise for differential expression miRNAs and potential target mRNAs by Pearson correlation.

## **Reviewers’ comments**

### **Reviewer’s report 1: Neil R Smalheiser, University of Illinois at Chicago, USA**

#### ***Reviewer summary***

This paper suggests that several miRNAs, notably mir-218-3p, are involved in E. Coli resistance in Meishan piglets, via targeting DLG5 among others. The finding is interesting but the ms. needs far better description of methods, and better analysis and presentation of the data. The fact that the experiment was based on only 3 animals per group is troubling!

#### ***Reviewer recommendations to authors***

1/Since most readers may be unfamiliar with Chinese pig strains, I suggest that you discuss in more detail why you chose Meishan piglets as a model system.

Author’s response: *Thanks for your suggestion. We added some description about Meishan pigs in discuss section: “Taihu pig is one breed with the highest reproductive traits in Chinese and foreign pigs around the world. Thereinto, Meishan is the most prominent representative population of Taihu pigs and has the advantage of higher litter size, more delicious meat, etc. Furthermore, compared to commercial western pig breeds, the Chinese Meishan pigs exhibit not only the higher litter size but also an increased physiological maturity which is correlated with the piglet survival rate before and at birth.”*

2/The Methods need to describe much more clearly how you challenged the piglets and what time lag between your challenge and the tissue extraction for miRNA levels. If I am correct, you have no way of telling in advance which piglets will be resistant? Sounds like you isolated duodenum during active infectious phase, which might complicate the pattern in the susceptible piglets? That should be discussed as possibly affecting your results.

Author’s response: *According to your requirement, we added detailed description about the method of challenging the piglets in our manuscript. As following:*

*“We selected three litters of weaning piglets at 35 days of age, 12 piglets per litter, with almost same birth weight and weaning weight. Twelve piglets per litter were randomly divided into two groups: the control group (two piglets) and the experimental group (ten piglets). Each piglet was housed individually in separate pens. They were fed ad libitum with a commercial-type compound feed for weaned piglets containing 21.7% crude protein, without antimicrobial additives and organic acids. Beginning at day 3 post-weaning, experimental piglets were challenged with a daily dose of 4.6×10* ^*8*^ *CFU of E. coli F18 strain once a day for up to 10 days, or until they showed diarrhea. No additional food was given and we ensured that piglets ate all food before the challenge experiment. Throughout the experiment, fecal shedding of the inoculated bacteria was monitored by daily fecal sampling and feces consistency was scored using the parameters “normal”, “pasty” and “watery”. Only piglets with watery feces were considered as diarrheic. The intestinal tracts of the diarrheic pigs were used to carry out a series of experiments, such as E. coli F18 bacteria counting, histopathological detection and adhesion test of the pathogens to the epithelial cells of small intestine in vitro [48].”*

*For the identification and selection of E. coli F18-resistant and –susceptible piglets, we mainly used the following method:*

*“Nine piglets showing “watery diarrhea” were defined as the “diarrhea group” and eight normal piglets as the “normal group”. After slaughter, we took intestinal tissues (duodenum, jejunum and ileum) to detect bacterial numbers. In all piglets detected by the binding assay, we strictly identified piglets displaying no adherence with F18-expressing fimbriae of the standard ETEC strain as E. coli F18-resistant individuals (Additional file 6A). In contrast, piglets displaying a large amount of adherence were identified as E. coli F18-susceptible individuals (Additional file 6B and 6C).”*

***Additional file 6. Adhesion test for intestinal epithelial cells for E. coli F18-resistant and -sensitive piglets.*** *The adhesion of Escherichia coli F18 to intestinal epithelial cells in Meishan piglets, A represents F18-resistant piglets displaying no adherence with F18-expressing fimbriae of the standard ETEC strain; B represents F18ab-susceptible piglets displaying a large amount of adherence with F18ab-expressing fimbriae of the standard ETEC strain, C represents F18ac-susceptible piglets displaying a large amount of adherence with F18ac-expressing fimbriae of the standard ETEC strain. Photos were taken with an oil immersion lens at 1000× magnification.*

*48. Liu L, Wang J, Zhao QH, Zi C, Wu ZC, Su XM, et al. Genetic variation in exon 10 of the BPI gene is associated with Escherichia coli F18 susceptibility in Sutai piglets. Gene. 2013;523:70–75.*

3/When only 3 animals per group are studied, there is a very high risk of false-positive findings, even with 2-fold elevations as seen here. I strongly urge you to carry out permutation analysis, i.e. take the set of 6 animals and randomly assort them to two groups (n=3 in each group) in all combinations. Do you find a similar number of 2-fold changes in miRNAs in all permutations, or only in the case where all 3 samples are susceptible and the other 3 are resistant? The traditional p-values as done here are not sufficient to ensure that the data are robust.

Author's response: *In our study, we performed a comparative microRNA transcriptome study on duodenum tissues between Meishan sensitive group (n=3) and resistant group (n=3) using Illumina Solexa sequencing technology. For high-throughput sequencing, generally speaking, 3 repeats meet the requirements of sequencing analysis. Besides, most studies also have 3 biology repeat in sample processing (Bai et al., 2014; Chen et al., 2012; Tao et al., 2013).*

*Bai Y, et al. (2014) A comprehensive microRNA expression profile of the backfat tissue from castrated and intact full-sib pair male pigs. BMC Genomics 2014, 15:47.*

*Chen C, et al. (2012) Solexa Sequencing Identification of Conserved and Novel microRNAs in Backfat of Large White and Chinese Meishan Pigs. PLoS ONE 7(2): e31426.*

*Tao X, Xu Z (2013) MicroRNA Transcriptome in Swine Small Intestine during Weaning Stress. PLoS ONE 8(11): e79343.*

*About the identification of differential miRNAs, we performed the comparison of the known miRNA expression between two groups (sensitive and resistant group) to find out the differentially expressed miRNAs. The expression of miRNA was shown in two samples by plotting Log2-ratio figure and Scatter Plot. Firstly, we normalized the expression of miRNA in two samples (sensitive and resistant group) to get the expression of transcript per million. When the normalized expression of a certain miRNA was zero between two samples, we revised its expression value to 0.01. While if the normalized expression of a certain miRNA was lower than 1, further differential expression analysis was conducted without this miRNA. Normalized expression (NE)=Actual miRNA count/Total count of clean reads×1000000. Moreover, we calculated the fold-change and P-value from the normalized expression. Then generate the log2ratio plot and scatter plot. Fold_change=log2(resistant group/sensitive group).*

4/I would strongly urge you to examine another cohort of piglets and at least measure mir-218-3p and other altered miRNAs, to make sure that the changes are reproducible.

Author’s response: *Many thanks for your suggestion. In our study, we obtained some differential expression miRNAs related to E. coli F18 by Solexa sequencing and further verification was performed between F18-sensitive and resistant piglets by Stem-loop RT-qPCR. However, your advice is very reasonable. In the next step work, we will detect the expression in different populations of piglets and analyze the function of miRNAs and target genes by knockdown or overexpression at the cellular level. If we achieve some breakthrough results, we hope to continue to get your guide.*

5/You appear to have isolated duodenum and jejunum, and measured target mRNAs in both tissues, but did not measure jejunum for miRNAs?? Why not? Especially, it would help to measure mir-218-3p and other altered miRNAs in jejunum.

Author’s response: *Escherichia coli (E. coli) are a group of gram negative flagellated bacteria that normally reside and multipl\y in the intestinal tract of all animals. Veterinary pathology experiments demonstrated that duodenum and jejunum are main place where E. coli F18 strain colonizes and replicates. The jejunum indeed can be used as samples for E.coli F18 adhesion test. In previous studies, duodenum was used for E.coli F18 adhesion and high-throughput sequencing (Bao, et al., 2012; Wu et al., 2016), in view of combination the data of previous high-throughput sequencing, we still choose the duodenum in this miRNA sequencing. However, we have isolated duodenum and jejunum for systematic qPCR validation.*

*Wu ZC, Liu Y, Dong WH, et al. CD14 in the TLRs signaling pathway is associated with the resistance to E. coli F18 in Chinese domestic weaned piglets. Scientific Reports, 2016, 6:24611.*

*Bao WB, Ye L, Pan ZY, et al. Microarray analysis of differential gene expression in sensitive and resistant pig to Escherichia coli F18[J]. Animal genetics, 2012, 43(5): 525–534.*

6/Some primary data are alluded to but should be explicitly presented. For example, you show p-values for correlation coefficients (r =), but you should show the r values themselves directly as well. In Table 1, with only 6 samples, you have room to display individual sample values and SDs in addition to the summary ratios and *p*-values. This would allow us to see if you have any high variability or outright outlier values in your data, which is crucial when there are only 3 samples per group.

Author’s response: *Thanks for your comments. We revised the Table 1 with adding the mean values and SDs. Table 1 shows the differential expression between E. coli F18-resistant group and E. coli F18-sensitive group by qRT-PCR. However, the comparison of miR-SEQ and qRT-PCR has been shown in Fig. 5.*

7/The authors should ideally demonstrate that mir-218-3p and DLG5 are both expressed in gut epithelial cells, which is not a given since they carried out measurements on entire duodenal tissue.

Author’s response: *In our manuscript, we performed a comparative miRNA sequencing of duodenal tissues between E. coli F18-resistant group and E. coli F18-sensitive group, and then we screened out some differential expression miRNAs including mir-218-3p. For qRT-PCR detection in duodenal tissues, our original purpose is to verify the result of miRNA sequencing. Meanwhile, we could further analyze the correlation of mir-218-3p and DLG5 expression.*

*However, the expert’s opinion is very reasonable. To analyze the function of DLG5 gene, we performed some on-going studies in gut epithelial cells. Actually, it extremely difficult to obtain pure epithelial cells (possibly include other intestinal cells). To avoid other intestinal cells, we strictly conducted the following sampling process: (1) after slaughter, the duodenum was incubated on ice for 1 h. Then, pre-cooling PBS (PMSF, NaN3) mixture washed the duodenum and exposed the intestinal inner-wall. (2) We scraped the intestinal mucosa with the slides, PBS washed it and centrifuged for 10 min at 200×g. This process is repeated 4 times until the clean precipitation. (3) We added NaHCO*_*3*_ *(NaN*_*3*_ *, PMSF) mixture and grinded 40 times, then centrifuged for 10 min at 450×g. (4) we proceeded a hung heavy precipitation with separation buffer (EDTA, NaN*_*3*_ *, PMSF), grinded 20 times, then centrifuged for 10 min at 300×g. This process is repeated 4 times. (5) Once again, we proceeded a hung heavy precipitation with Mg* ^*2+*^ *buffer (EDTA, NaN*_*3*_ *, PMSF), grinded 20 times, stewing at 4 °C for 30–60 min. (6) The supernate fluid was filtered by lanoline to remove the nucleus, then centrifuged for 10 min at 450×g. (7) Finally, proceeded a hung heavy precipitation with Final buffer (KH*_*2*_ *PO*_*4*_ *, Sorbitol, NaCl, NaN*_*3*_ *) and stored at −80 °C until further use. Meanwhile we further confirmed that the sample was only epithelial cells by microscopic examination, see below:*

#### ***Minor issues***

1/You say you “randomly selected” 15 miRNAs to verify by RT-PCR, but the list does not look random.

Author’s response: *Thanks for your advice. We have deleted the word “randomly” in our manuscript.*

2/The Discussion section talks about some methodological points that belong in Methods.

Author’s response: *About some methodological points, such as bacterial infection experiment, we emphasized the three key points to improve the effectiveness and feasibility of our piglet diarrhea model using an artificial challenge experiment.*

3/The paper talks about “target genes” which are really target mRNAs.

Author’s response: *“target genes” indeed seems a little absolute. We revised “target genes” as “potential target mRNAs”.*

#### ***Amended comments***

The manuscript is improved but I am rather confused and worried by the findings presented. They show that mir-218-3p is down-regulated in sensitive tissues by RT-PCR (Table 1), and DLG5 is also down-regulated in the same tissue (fig. 8), yet they assert that the two are strongly NEGATIVELY correlated (fig. 9). That does not seem right to me! If the two are indeed negatively correlated [across both sensitive and resistant tissues], then one of the two should be UP-regulated in sensitive tissues. It seems that there is a fundamental discrepancy between sequencing data (fig. 5) which shows that mir-218 is UP-regulated in sensitive tissues and the RT-PCR data (Table 1) which shows a significant DOWN-regulation in the same tissue, thus not a validation but instead in opposition. Something is not right, and it is crucial to clarify these before publication.

Author’s response: *Many thanks for your pointing out mistakes. We thought the order of miR-name (from Table 1, as follow) was consistent with the detected miRNA (from Original data, as follow). We mistakenly copied original data into Table 1. We felt very ashamed for such a mistake. We revised the Table 1 according to the original data.*

***Table 1.*** *Validation of the miR-SEQ expression profiles of selected miRNAs by qRT-PCR.*

**Table:** 

***miR-name***	***Accession No.***	***E. coli F18-resistant group***	***E. coli F18-sensitive group***	***P-value***
*ssc-miR-499-5p*	*MIMAT0013877*	*1.397±0.680*	*0.814±0.154*	0.041*
*ssc-miR-676-5p*	*MIMAT0017382*	*0.808±0.408*	*1.538±0.727*	*0.163*
*ssc-miR-432-3p*	*MIMAT0017384*	*0.649±0.443*	*1.901±0.553*	*0.215*
*ssc-miR-196b*	*MIMAT0013923*	*2.275±0.991*	*0.478±0.079*	0.036*
*ssc-miR-421-5p*	*MIMAT0017970*	*2.370±1.109*	*0.467±0.055*	*0.127*
*ssc-miR-202-5p*	*MIMAT0013948*	*0.815±0.508*	*1.404±0.044*	*0.133*
*ssc-miR-885-3p*	*MIMAT0013903*	*0.593±0.183*	*0.378±0.011*	*0.180*
*ssc-miR-493-5p*	*MIMAT0025377*	*1.830±1.344*	*0.821±0.563*	*0.146*
*ssc-miR-218-3p*	*MIMAT0017969*	*1.890±1.181*	*0.650±0.294*	0.012*
*ssc-miR-155-3p*	*MIMAT0017953*	*1.284±0.492*	*0.859±0.269*	*0.280*
*ssc-miR-208b*	*MIMAT0013912*	*0.427±0.127*	*0.559±0.112*	*0.130*
*ssc-miR-187*	*MIMAT0020587*	*0.582±0.224*	*1.939±0.850*	*0.231*
*ssc-miR-450b-3p*	*MIMAT0017380*	*0.817±0.442*	*1.659±0.823*	*0.171*
*ssc-miR-136*	*MIMAT0002158*	*1.878±1.322*	*0.695±0.332*	0.045*
*ssc-miR-424-3p*	*MIMAT0013921*	*0.742±0.234*	*1.625±0.926*	*0.215*

* p < 0.05.

### **Reviewer’s report 2: Weixiong Zhang, Washington University, USA**

#### ***Reviewer summary***

The manuscript described a study profiling miRNAs in Chinese domestic weaned piglets challenged with E. coli F18 strain which is know to cause porcine post-weaning diarrhea. Two small RNA libraries were constructed using 3 pooled samples of F18-resistant groups and 3 pooled samples of F18-sensitive groups, and sequenced by the Illumina deep sequencing platform. Fifteen upregulated and 9 downregulated miRNAs were identified between the two groups of piglets, and their potential mRNA target genes were identified using results in the Miranda database. The expression of some of the miRNAs and their target genes were experimentally validated using PCR assays. GO enrichment and KEGG pathway analyses were performed on the target genes to assess biological relevance and significance of the results. The overall design of the study and the profiling experiments are sound. The results of differentially expressed miRNAs that are potentially responsive to F18 infection may be valuable for future studies and to practitioners in a focused area. The overall approach taken is conventional and is not novel.

#### ***Reviewer recommendations to authors***

Two major areas of improvement can be introduced to improve the quality and expand the scope of the study. The first is to identify novel miRNAs using the sequencing data. This can be done using many published methods, e.g., miRDeep. (Or write to me, weixiong.zhang@wustl.edu and I am happy to provide our miRvial tool for miRNA prediction.) The novel miRNAs that are potentially specifically responsive to F18 infection may provide deep insights to miRNA gene regulation. The second area of improvement is to introduce mRNA profiling using RNA-seq. Ideally such data can be gathered using the same total RNA that was used to profile miRNAs. Profiling of mRNA gene expression can be integrated with the miRNA target information to paint a genome-wide picture of miRNA-mRNA regulation. Another possible improvement is not to pool the 3 samples into one library, but rather separate them using barcodes and profile them using multiplexing sequencing. The new data can provide some statistical power in calling differentially expressed miRNAs. In light of these possible improvements, which can be easily incorporated, the current study seemed rather rudimentary.

Author’s response: *Many thanks for your valuable suggestions. Firstly, for novel miRNAs, we added the identification of novel miRNAs by mireap software. As following: “To identify novel miRNA genes among the unannotated sequences in our libraries, we employed the mireap program (https://sourceforge.net/projects/mireap/), which processes high-throughput sequencing data sets.” was placed in th8e section “Sequencing data analysis” of “Methods”. Finally, we identified 681 novel miRNAs, as shown in added “Additional file 2”.*

*Secondly, your advice on mRNA profiling is very reasonable. In our manuscript, we have considered the combination of mRNA and miRNA. In previous studies, we have obtained differential expression genes (DEGs) between Meishan F18-resistant and -sensitive groups (Samples are in complete accord with this miRNA study) by mRNA transcriptome sequencing (data are available at NCBI’s SRA, PRJNA271310). In this study, we also found some differential miRNAs and their potential target genes were predicted, so we further screened out important target genes based on previous DEGs. Moreover, about the samples for sequencing, we did not pool the 3 samples into one library, but six small RNA libraries were constructed from resistant (R1, R2, R3) and sensitive (S1, S2, S3) piglets to E. coli F18, respectively. We will systematically perform an in-depth study of miRNAs and target genes in future, and then hope to continue to get your guidance.*

#### ***Minor issues***

The quality of some of the figures, e.g., Fig 6, should be improved. It’s difficult to see the content of Fig 6 and Fig 7.

Author’s response: *According to your requirements, we have improved the resolution of all figures to 500 dpi.*

#### ***Amended comments***

The authors made their effort to answer the reviewers’ questions and comments. However, the additional work they have done didn’t seem to improve the quality of the work. In particular, the authors added the result on novel miRNAs they could predict using an off-the-shelf method on their sequencing data. However, they didn’t integrate the result to help achieve their goal of identifying miRNA gene regulators in the process of E. coli infection. So the additional work of novel miRNA prediction is completely useless to the current study and should be removed if they indeed didn’t want to add any functional information of the novel miRNAs. Second, while in their responses to reviewers’ comments they mentioned that they used their previous mRNA data in the revision, no where in the new manuscript such results or description can be found. In their responses, they indicated that they in fact would like to defer integration of miRNA and mRNA data to a future study. In short, the revision is essentially the same as the original submission, and as such I don’t believe it meet the standard of the journal.

Author’s response: *Many thanks for your comments. Referring to some literature, most studies mainly focused on the predicted novel miRNAs, its free energy and stem-loop structure. Therefore, based on the predicted novel miRNAs, we further analyzed the typical stem-loop structure and free energy. Revision in manuscript as following:*

*In the section “* ***Sequencing data analysis*** *” form “* ***Methods*** *”, we added “The RNAfold software in the ViennaRNA Package 2.0 [20] was also used to predict the typical secondary structures of the miRNA precursors.” In the “* ***Differential miRNAs in E. coli F18-sensitive and -resistant Meishan piglets*** *” from “* ***Results*** *”, we added “In addition, 681 potential novel miRNA candidates were obtained from RG and SG libraries. These pre-miRNAs possessed a typical stem-loop structure and free energy ranging from −64.8 Kcal/mol to −18.2 Kcal/mol (Additional file 3). The folding (free energy>50.0 Kcal/mol) are shown in Additional file 4.*

***Additional file 4: Partial secondary structure of novel microRNAs.*** *Folding secondary structure of novel microRNAs and flanking sequences was predicted by RNAfold. The entire sequence represents pre-miRNAs.*

*In our study, the original aim is to identify some known miRNAs related to E. coli F18 infection, which is the main point and meaning of this manuscript. As you said, we simply predicted the novel miRNAs and not analyze their function. It is nearly impossible for analyzing the function of all novel miRNAs, and we think that these novel miRNAs probably provide some database information on pig miRNAs, which aimed to provide useful information for future study of other researchers. Because you are an authoritative expert in this field, our study is relatively preliminary and we hope to get your understanding.*

*About the integration of miRNA and mRNA data, our manuscript previous included these contents. In the section “* ***Predicted target genes and functional annotation*** *” from “* ***Methods*** *”, we have mentioned “According to previous differential expression genes (DEGs) between Meishan F18-resistant and -sensitive groups by transcriptome sequencing (data are available at NCBI’s SRA, PRJNA271310), we further selected potential target mRNAs related to E. coli F18 infection. On this basis, the regulatory network of DEMs and potential target mRNAs was established by Cytoscape software [24].”. In the section “* ***miRNA target gene prediction, GO enrichment and KEGG pathway analysis*** *” from “* ***Results*** *”, we have mentioned “Based on previous DGEs and functional enrichment, we further screened out important target genes related to E. coli F18 infection by Venny software (http://bioinfogp.cnb.csic.es/tools/venny/) and analyzed the regulatory network between important target genes and differential miRNAs (Fig. 7). Based on our results, we speculate that the α-(1, 2) fucosyltransferase 2 gene (FUT2) and Discs, large homolog 5 (DLG5) genes were the targets of down-regulated ssc-miR-218-3p, the MUC4 gene was the target of down-regulated ssc-miR-136, MyD88 was the target of up-regulated ssc-miR-499-5p, LBP and Toll-like receptor (TLR4) genes were the target of up-regulated ssc-miR-196b.”.*

## Additional files

Additional file 1: Information of known miRNAs in the duodenum of Meishan piglets. (XLS 107 kb)
Additional file 2: Differential expression of known miRNAs in duodenal tissues between *E. coli* F18-resistant and -sensitive groups. (DOC 48 kb)
Additional file 3: Information of novel miRNAs in the duodenum of Meishan piglets. (XLSX 105 kb)
Additional file 4: Partial secondary structure of novel microRNAs. Folding secondary structure of novel microRNAs and flanking sequences was predicted by RNAfold. The entire sequence represents pre-miRNAs. (JPG 102 kb)
Additional file 5: Potential target genes prediction analysis of differential expressed miRNAs between *E. coli* F18-resistant and -sensitive groups. (XLSX 2596 kb)
Additional file 6: Gene ontology classification annotated by DAVID for potential target genes of differentially expressed miRNAs. The figure shows partial GO enrichment for the predicted target genes from ontologies of biological processes, cellular component and molecular function. (JPG 1078 kb)
Additional file 7: Adhesion test for intestinal epithelial cells for *E. coli* F18-resistant and -sensitive piglets. The adhesion of *Escherichia coli* F18 to intestinal epithelial cells in Meishan piglets, A represents F18-resistant piglets displaying no adherence with F18-expressing fimbriae of the standard *ETEC* strain; B represents F18ab-susceptible piglets displaying a large amount of adherence with F18ab-expressing fimbriae of the standard *ETEC* strain, C represents F18ac-susceptible piglets displaying a large amount of adherence with F18ac-expressing fimbriae of the standard *ETEC* strain. Photos were taken with an oil immersion lens at 1000× magnification. (JPG 323 kb)
Additional file 8: Primer sequences for real-time PCR. (DOC 38 kb)
Additional file 9: Real-time PCR primer information of target genes. The selected genes were identified by real-time PCR. The housekeeping genes, *GAPDH*, *TBP1* and *ACTB* were used as the internal controls. The data were analyzed by the cycle threshold (C(t)) method. (DOC 40 kb)

## Abbreviations

PWD: Post-weaning diarrhea
*E. coli*: *Escherichia coli*
3′UTR: 3′untranslated region
ETEC: Enterotoxigenic *Escherichia coli*
LPS: Lipopolysaccharide
*FUT1*: Alpha (1,2)fucosyltransferase
RG: Resistant groups
SG: Sensitive groups
*DLG5*: Discs, large homolog 5
