# Supplementary material for: Identification of microRNAs regulating Escherichia coli F18 infection in Meishan weaned piglets
Source: Biol Direct. 2016 Nov 3;11:59. doi: 10.1186/s13062-016-0160-3 (PMC5093996; doi:10.1186/s13062-016-0160-3)
Supplement: Additional file 2: — Differential expression of known miRNAs in duodenal tissues between E. coli F18-resistant and -sensitive groups. (DOC 48 kb) [file 13062_2016_160_MOESM2_ESM.doc]

**Table S4.** Differential expression of known miRNAs in duodenal tissues between *E. coli* F18 resistant and sensitive groups.

| **miR-Name** | **Mature sequence (5'→3')** | **Resistant group (R)** | **Sensitive group (S)** | **Fold-Change (log2R/S)** | **Expression Level** |
| --- | --- | --- | --- | --- | --- |
| ssc-miR-4334-5p | cccuggagugacgggggug | 0.223015466 | 0.820496948 | -1.879354206 | down |
| ssc-miR-218-3p | augguucugucaagcaccaug | 0.747183022 | 2.039339339 | -1.448568276 | down |
| ssc-miR-155-3p | uccuacauguuagcauuaaca | 0.23459932 | 0.633227259 | -1.432524531 | down |
| ssc-miR-208b | auaagacgaacaaaagguuugu | 0.23459932 | 0.609421195 | -1.377240846 | down |
| ssc-miR-187 | ucgugucuuguguugcagccgg | 2.577642164 | 6.430923217 | -1.318973866 | down |
| ssc-miR-4337 | aggguauauaagccuucacugg | 0.457614785 | 1.093622935 | -1.256909833 | down |
| ssc-miR-450b-3p | uugggaacauuuugcauccau | 0.680630251 | 1.577824675 | -1.212993725 | down |
| ssc-miR-136 | acuccauuuguuuugaugaugga | 25.92641529 | 54.89911458 | -1.082360135 | down |
| ssc-miR-424-3p | caaaacgugaggcgcugcuau | 10.39733878 | 21.28307055 | -1.033491992 | down |
| ssc-miR-493-5p | uuguacaugguaggcuuucauu | 7.533268179 | 3.749513452 | 1.00657249 | up |
| ssc-miR-7136-3p | ucucaguguuugaaccagaagc | 1.427813273 | 0.695277493 | 1.038146525 | up |
| ssc-miR-7141-5p | gacgguuuggacguuaagaac | 0.457614785 | 0.211075753 | 1.116372805 | up |
| ssc-miR-885-3p | aggcagcgggguguaguggau | 0.46919864 | 0.211075753 | 1.152437969 | up |
| ssc-miR-4331 | uguggcugugguguaggccagc | 272.6519812 | 121.5724648 | 1.165244132 | up |
| ssc-miR-202-5p | uuccuaugcauauacuucuuu | 17.41980882 | 7.524130095 | 1.211132092 | up |
| ssc-miR-7140-5p | caacucaagggggcaucauuca | 3.368210248 | 1.3035797 | 1.369503405 | up |
| ssc-miR-421-5p | ccucauuaaauguuuguugaauga | 0.747183022 | 0.273125987 | 1.451895086 | up |
| ssc-miR-7136-5p | ucugguccagacacuguggagc | 12.28314573 | 4.022639439 | 1.610465748 | up |
| ssc-miR-7135-5p | ucucugagacacugacugugg | 8.367031881 | 2.484177921 | 1.751947427 | up |
| ssc-miR-1249 | acgcccuucccccccuucuuca | 67.2011119 | 19.51350024 | 1.784012519 | up |
| ssc-miR-196b | uagguaguuuccuguuguuggg | 5.795479871 | 1.539580505 | 1.912390814 | up |
| ssc-miR-432-3p | uggauggcuccuccauggcu | 1.193213953 | 0.273125987 | 2.127214261 | up |
| ssc-miR-676-5p | cucuucaaucucaggacucgca | 0.981782342 | 0.211075753 | 2.217642357 | up |
| ssc-miR-499-5p | uuaagacuugcagugauguuu | 2374.367622 | 367.0070913 | 2.693663479 | up |
