# Supplementary figures and images for: Identification of microRNAs regulating Escherichia coli F18 infection in Meishan weaned piglets
Source: Biol Direct. 2016 Nov 3;11:59. doi: 10.1186/s13062-016-0160-3 (PMC5093996; doi:10.1186/s13062-016-0160-3)

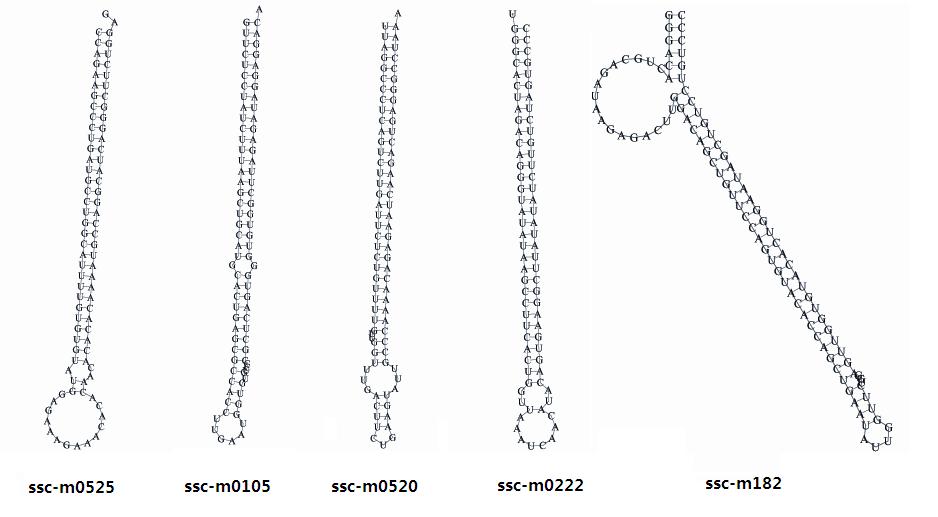

Supplement: Additional file 4: — Partial secondary structure of novel microRNAs. Folding secondary structure of novel microRNAs and flanking sequences was predicted by RNAfold. The entire sequence represents pre-miRNAs. (JPG 102 kb) [file 13062_2016_160_MOESM4_ESM.jpg]

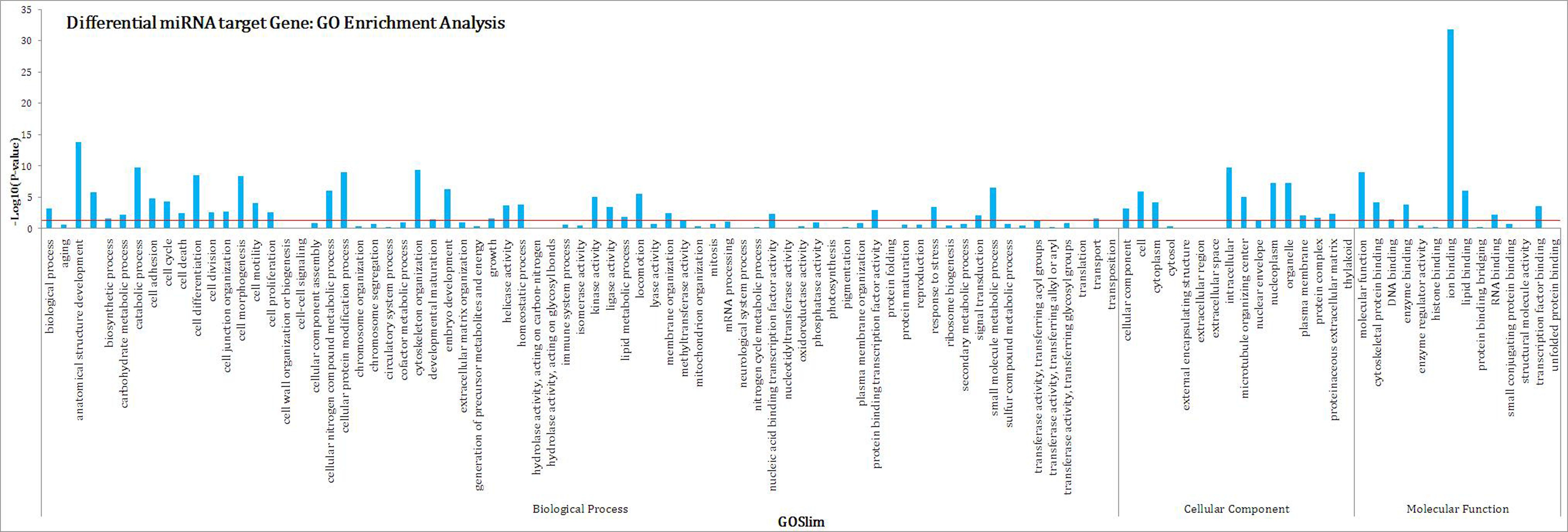

Supplement: Additional file 6: — Gene ontology classification annotated by DAVID for potential target genes of differentially expressed miRNAs. The figure shows partial GO enrichment for the predicted target genes from ontologies of biological processes, cellular component and molecular function. (JPG 1078 kb) [file 13062_2016_160_MOESM6_ESM.jpg]

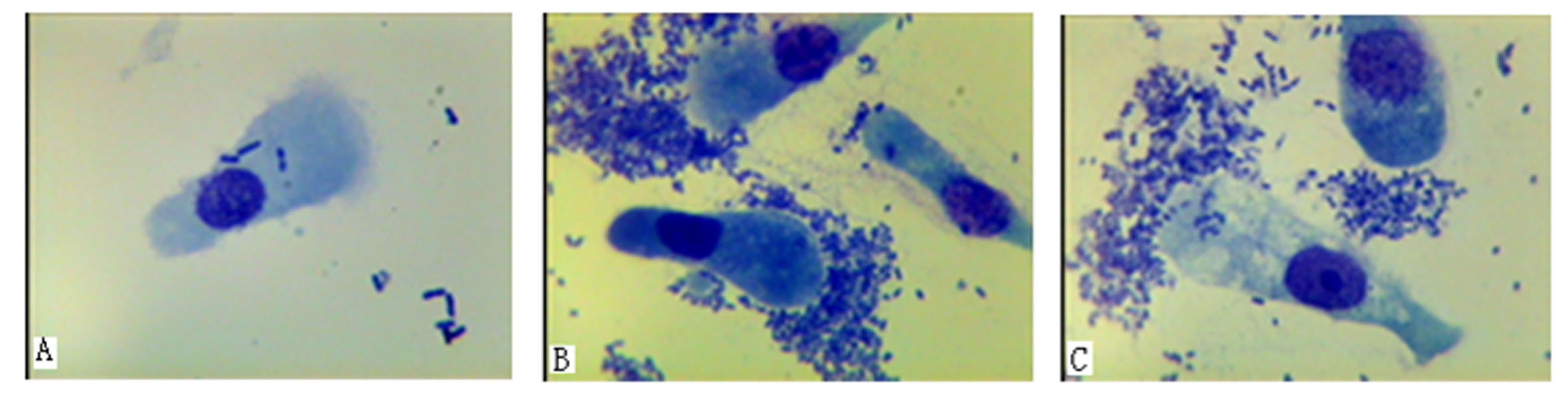

Supplement: Additional file 7: — Adhesion test for intestinal epithelial cells for E. coli F18-resistant and -sensitive piglets. The adhesion of Escherichia coli F18 to intestinal epithelial cells in Meishan piglets, A represents F18-resistant piglets displaying no adherence with F18-expressing fimbriae of the standard ETEC strain; B represents F18ab-susceptible piglets displaying a large amount of adherence with F18ab-expressing fimbriae of the standard ETEC strain, C represents F18ac-susceptible piglets displaying a large amount of adherence with F18ac-expressing fimbriae of the standard ETEC strain. Photos were taken with an oil immersion lens at 1000× magnification. (JPG 323 kb) [file 13062_2016_160_MOESM7_ESM.jpg]
