# Supplementary material for: Identification of microRNAs regulating Escherichia coli F18 infection in Meishan weaned piglets
Source: Biol Direct. 2016 Nov 3;11:59. doi: 10.1186/s13062-016-0160-3 (PMC5093996; doi:10.1186/s13062-016-0160-3)
Supplement: Additional file 8: — Primer sequences for real-time PCR. (DOC 38 kb) [file 13062_2016_160_MOESM8_ESM.doc]

**Table S1.** Primer sequences for real-time PCR.

| **Mature miRNA ID** | **Forward primer (5'→3')** | **Annealing temperature (℃)** | **Products size (bp)** |
| --- | --- | --- | --- |
| ssc-miR-218-3p | GGGGATGGTTCTGTCAAGC | 60 | 100 |
| ssc-miR-155-3p | GGGGGTCCTACATGTTAGCA | 60 | 63 |
| ssc-miR-208b | GGGGGATAAGACGAACAAAAG | 60 | 64 |
| ssc-miR-187 | GGGTTCGTGTCTTGTGTTGC | 60 | 65 |
| ssc-miR-450b-3p | GGGATTGGGAACATTTTGC | 60 | 64 |
| ssc-miR-136 | GGGGACTCCATTTGTTTTGAT | 60 | 63 |
| ssc-miR-424-3p | GGGACAAAACGTGAGGCG | 60 | 65 |
| ssc-miR-493-5p | GGGGTTGTACATGGTAGGCT | 60 | 63 |
| ssc-miR-885-3p | GAAAGGCAGCGGGGTGTA | 60 | 64 |
| ssc-miR-202-5p | GGGGGGATTCCTATGCATAT | 60 | 62 |
| ssc-miR-421-5p | GCCCCCTCATTAAATGTTTGT | 60 | 66 |
| ssc-miR-196b | GGGGGTAGGTAGTTTCCTGTT | 60 | 66 |
| ssc-miR-432-3p | GGGGTATGGATGGCTCCTC | 60 | 65 |
| ssc-miR-676-5p | GGGGCTCTTCAATCTCAGGA | 60 | 64 |
| ssc-miR-499-5p | GGGGGTTAAGACTTGCAGTG | 60 | 64 |
| U6(F) | TCGCTTTGGCAGCACCTAT | 60 | 64 |
| U6(R) | AATATGGAACGCTTCGCAAA | 60 | 64 |
