# Supplementary material for: Identification of microRNAs regulating Escherichia coli F18 infection in Meishan weaned piglets
Source: Biol Direct. 2016 Nov 3;11:59. doi: 10.1186/s13062-016-0160-3 (PMC5093996; doi:10.1186/s13062-016-0160-3)
Supplement: Additional file 9: — Real-time PCR primer information of target genes. The selected genes were identified by real-time PCR. The housekeeping genes, GAPDH, TBP1 and ACTB were used as the internal controls. The data were analyzed by the cycle threshold (C(t)) method. (DOC 40 kb) [file 13062_2016_160_MOESM9_ESM.doc]

**Table S2.** Real-time PCR primer information of target genes

| Gene | Accession number | Primers | Length of amplified  fragment (bp) |
| --- | --- | --- | --- |
| *DLG5* | XM_005671132 | F: 5′- ATCCCTCTGTCATCGACCCA-3′ | 185 |
|  |  | R: 5′- GTGCAGGTTCCCACCACATA-3′ |  |
| *FUT2* | U70881.2 | F: 5′-AATCCCTGACCTCACTCCGTG-3′ | 123 |
|  |  | R: 5′-CGGAACTACAACTGCTGGCC-3′ |  |
| *MUC4* | DQ848681.1 | F: 5'-GGCCCACCTTAAGATTCCCA-3' | 131 |
|  |  | R: 5'-GCTTCTCCTTAGCATGCCCAG-3' |  |
| *LBP* | NM_001128435 | F: 5′-ATATCGAATCTGCGCTCCGA-3′ | 136 |
|  |  | R: 5′-TTGATGCCAACCATTCTGTCC-3′ |  |
| *MyD88* | EU056736 | F: 5'-GTGCCGTCGGATGGTAGT -3' | 173 |
|  |  | R: 5'-CAGTGATGAACCGCAGGAT-3' |  |
| *TLR4* | AB232527 | F: 5'-CAGATAAGCGAGGCCGTCATT-3' | 113 |
|  |  | R: 5′-5'-TTGCAGCCCACAAAAAGCA-3' |  |
| *GAPDH* | AF017079 | F:5′-ACATCATCCCTGCTTCTACCGG-3′ | 188 |
|  |  | R: 5′-CTCGGACGCCTGCTTCAC-3′ |  |
| *TBP1* | DQ845178.1 | F: 5′-AACAGTTCAGTAGTTATGAGC-3′ | 153 |
|  |  | R: 5′-AGATGTTCTCAAACGCTTCG-3′ |  |
| *ACTB* | NC_010445.3 | F: 5'-GTCGTACTCCTGCTTGCTGAT-3' | 119 |
|  |  | R: 5'CCTTCTCCTTCCAGATCATCGC-3' |  |

The selected genes were identified by real-time PCR. The housekeeping genes, *GAPDH*, *TBP1* and *ACTB* were used as the internal controls. The data were analyzed by the cycle threshold (C(t)) method.
